# Transplantation of hybrid adipose-derived stem cell sheet with autologous peritoneum: An *in vivo* feasibility study

**DOI:** 10.1016/j.heliyon.2023.e12992

**Published:** 2023-01-14

**Authors:** Natsuki Matsuo, Takeshi Ohki, Shota Aoyama, Shigeki Yamaguchi, Michio Itabashi, Hiroto Egawa, Masakazu Yamamoto

**Affiliations:** a Department of Surgery, Institute of Gastroenterology, Tokyo Women's Medical University, 8-1 Kawada-Cho Shinjuku-ku Tokyo, 162-8666, Japan; b Department of Advanced Biomedical Engineering and Science, Tokyo Women's Medical University, 8-1 Kawada-Cho Shinjuku-ku Tokyo, 162-8666, Japan; c Department of Gastroenterological Surgery, Utsunomiya Memorial Hospital, 1-3-16 Ohdori Utsunomiya-shi Tochigi, 320-0811, Japan

**Keywords:** Adipose-derived stem cell sheet, Hybrid adipose-derived stem cell sheet, Peritoneum, Anastomotic leakage, Temperature-responsive culture dishes, Cell sheet engineering, ASC, Adipose-derived stem cell, HASC, Hybrid adipose-derived stem cell, MSC, Mesenchymal stem cell, SD rats, Sprague Dawley rats, SD-Tg rats, Sprague Dawley transgenic rats, PIPAAm, Poly N-isopropylacrylamide, TCPS, Tissue culture polystyrene surface

## Abstract

**Introduction:**

In regenerative medicine, cell sheet engineering has various advantages, including the retention of cells at the transplantation site for a longer period and the local delivery of growth factors and cytokines. Adipose-derived stem cell (ASC) is widely used owing to their various functions such as wound healing, immunomodulation, and nerve regeneration, in addition to their ability to differentiate into adipocytes, chondrocytes, and osteoblasts. ASC sheet generated using cell sheet engineering is considered effective in preventing anastomotic leakage, a serious postoperative complication in gastrointestinal surgery. However, the ASC sheet is too soft, thin, and brittle to handle with laparoscopic forceps during the operation. Therefore, we considered using the peritoneum, which is stiff and easy to collect while operating, as an alternative support. In this study, we explored the feasibility of using the peritoneum as a support for the precise transplantation of ASC sheets during surgery.

**Methods:**

ASCs were isolated from the subcutaneous fat of the inguinal region of Sprague-Dawley (SD) transgenic rats expressing green fluorescent protein. ASCs were cultured until passage 3, seeded in temperature-responsive culture dishes, and the resulting ASC sheet was harvested at more than 80% confluency. Non-transgenic SD rats were used for transplant experiments. The wall peritoneum was harvested from SD rats following laparotomy, and hybrid adipose-derived stem cell (HASC) sheet was prepared by laminating the peritoneum with ASC sheet. The cell sheets were transplanted on the backs of SD rats following the incision. On post-transplantation days 3 and 7, the specimens were extracted. ASC and HASC sheets were then compared macroscopically and histopathologically.

**Results:**

HASC sheet transplantation was macroscopically and histopathologically more effective than ASC sheet transplantation. The peritoneum provided sufficient stiffness as a support for precise transplantation.

**Conclusion:**

The newly developed HASC sheet, which combine the advantages of ASC sheet with those of the peritoneum, could be more useful for clinical application than the ASC sheet alone.

## Highlights

• Adipose-derived stem cell (ASC) sheet is effective for wound healing.
• ASC sheet is difficult to transplant with laparoscopic forceps.
• The peritoneum is relatively rigid and can be used as a support.
• Feasibility of hybrid adipose-derived stem cell (HASC) sheet composed of ASC sheet and peritoneum was evaluated.
• HASC sheet was easier to transplant than ASC sheet alone.

## Graphical abstract

**Figure:**
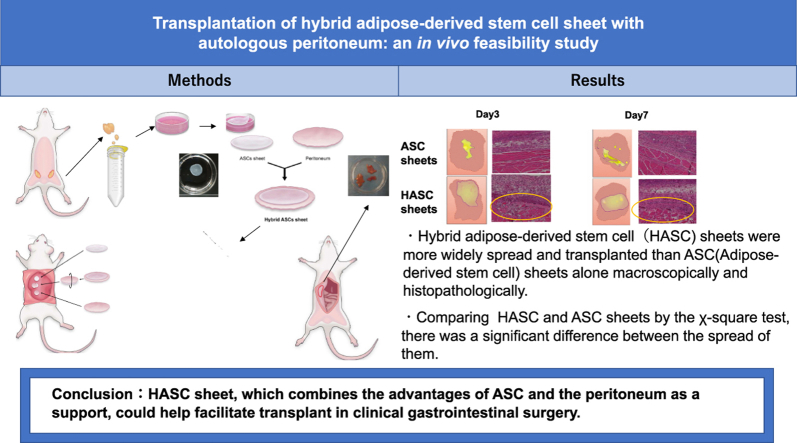

## 1 Introduction

The field of regenerative medicine is constantly progressing and has developed to the stage of direct clinical application. Among recent developments is cell sheet engineering, which involves the use of temperature-responsive culture dishes [1,2]. Cell sheets have several advantages, including the retention of cells at the transplantation site for a longer period and local delivery of growth factors and cytokines [3]. Cell sheet engineering has also developed to the point of clinical application for therapeutic treatment [4] and clinical research of cell sheets is progressing in various medical fields. In the field of gastroenterology, we previously reported that the oral mucosal epithelial cell sheet promotes esophageal mucosal regeneration and can effectively prevent stenosis [5,6].

The human body contains stem cells in various tissues, which function to replenish cells when tissues are damaged. Mesenchymal stem cell (MSC) is pluripotent stem cell with the ability to self-renew and differentiate with little risk of tumor formation [7]. MSC is known to have various functions such as wound healing, immunomodulation, and nerve regeneration, in addition to their ability to differentiate into adipocytes, chondrocytes, and osteoblasts [8]. Adipose-derived stem cell (ASC) offers several advantages and is widely used in regenerative medicine. ASC is functionally similar to MSC derived from the bone marrow. Moreover, ASC are minimally invasive, easy to harvest, can be obtained in large quantities, have a high proliferative capacity, and is considered to be less susceptible to cellular aging associated with proliferation. ASC secretes growth factors and cytokines that can promote wound healing [9,10] or improve the quality of tissues. Several studies have also reported cell sheet engineering methods using ASCs.

In recent years, laparoscopic approaches have become more common for lower gastrointestinal surgery. Robotic surgery is also becoming increasingly common for operation involving the lower rectum. Anastomotic leakage is one of the most serious complications of lower gastrointestinal surgery. Previous studies have reported the efficacy of ASC sheets transplanted at the anastomotic site in the lower gastric tract using animal models of anastomotic leakage; however, ASC sheets have not yet been applied in clinical practice [11]. Although effective transplantation is important to take advantage of the characteristics of ASC sheets during the surgery, current ASC sheets are too thin, soft, and brittle, and prone to shrinkage. Thus, it is difficult to transplant ASC sheets with laparoscopic forceps without support because the anastomotic site of colon or rectum is tubular rather than flat.

The peritoneum, which can be easily harvested during surgery, is a relatively rigid tissue; therefore, we hypothesized that it would serve as a useful support for ASC sheets for precise transplantation. Besides being rigid, the peritoneal membrane has physiological advantages, and since it is an autologous tissue graft, there should be no major immunological problems. Therefore, in this study, we developed hybrid adipose-derived stem cell (HASC) sheet by layering ASC sheet and the peritoneum and evaluated its feasibility using an animal model.

## 2 Materials & methods

### 2.1 Animals

The experimental procedures used for animals were approved annually by the Committee for Animal Research of Tokyo Women's Medical University (Approval numbers: AE19-122, AE20-096, and AE21-080). Forty male Sprague-Dawley (SD) rats, 8–11 weeks old and weighing 220–350 g (Japan SLC, Inc, Tokyo, Japan), were used for transplantation experiments. ASCs were isolated from 10 male SD-transgenic (SD-Tg) rats, 6–8 weeks old and weighing 165–280 g (Japan SLC, Inc), expressing enhanced green fluorescent protein. All rats were raised in individual cages with free access to water and food and were at kept at room temperature (22–24 °C; ∼45% relative humidity) under a 12-h light/dark cycle.

### 2.2 Experimental design

**Fig. 1:**
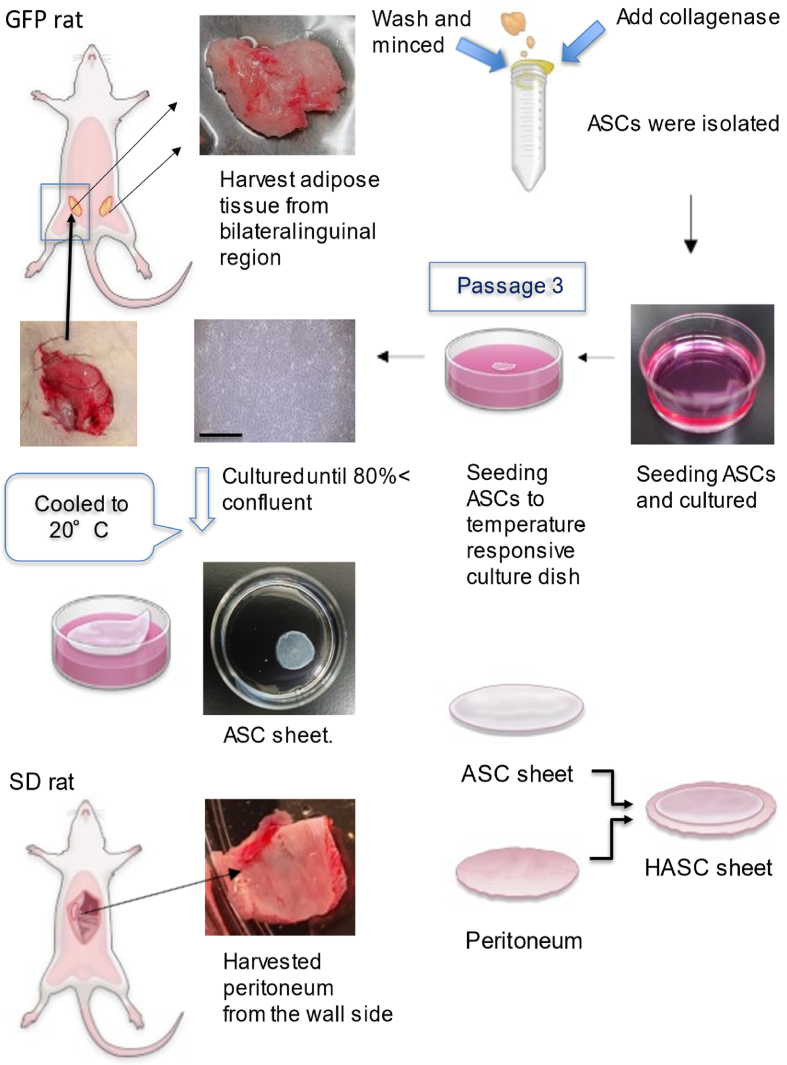
Preparation of adipose-derived stem cell (ASC) sheets and hybrid adipose-derived stem cell (HASC) sheets. First, the adipose tissue was harvested from the bilateral inguinal region of green fluorescent protein-expressing transgenic rats. Then, collagenase was added to the finely minced adipose tissue, and the tissue was shaken for 2 h and then separated using a cell strainer. ASCs pelleted by centrifugation were seeded in Dulbecco's modified Eagle medium supplemented with 10% fetal bovine serum. The cells were cultured and passaged upon reaching 80% confluency. At the third passage, the cells were seeded onto 35-mm temperature-responsive culture dishes and cultured in complete medium. After 24 h, the medium was changed, and ascorbic acid was added to the medium. The cells were cultured until reaching 90% confluency. Taking advantage of the characteristics of cell sheet engineering, the temperature-responsive culture dishes were placed in an incubator at 20 °C for 20 min to allow the cell sheets to detach spontaneously. The bottom row shows the process of making HASC sheet. Under inhalation anesthesia, the wall-side peritoneum of SD rats was removed. Since a cell sheet prepared in 35-mm temperature-responsive culture dishes were less than 30 mm in diameter, the excised peritoneum was trimmed into a circle of 35 mm diameter, and the ASC sheet was layered onto it.

**Fig. 2:**
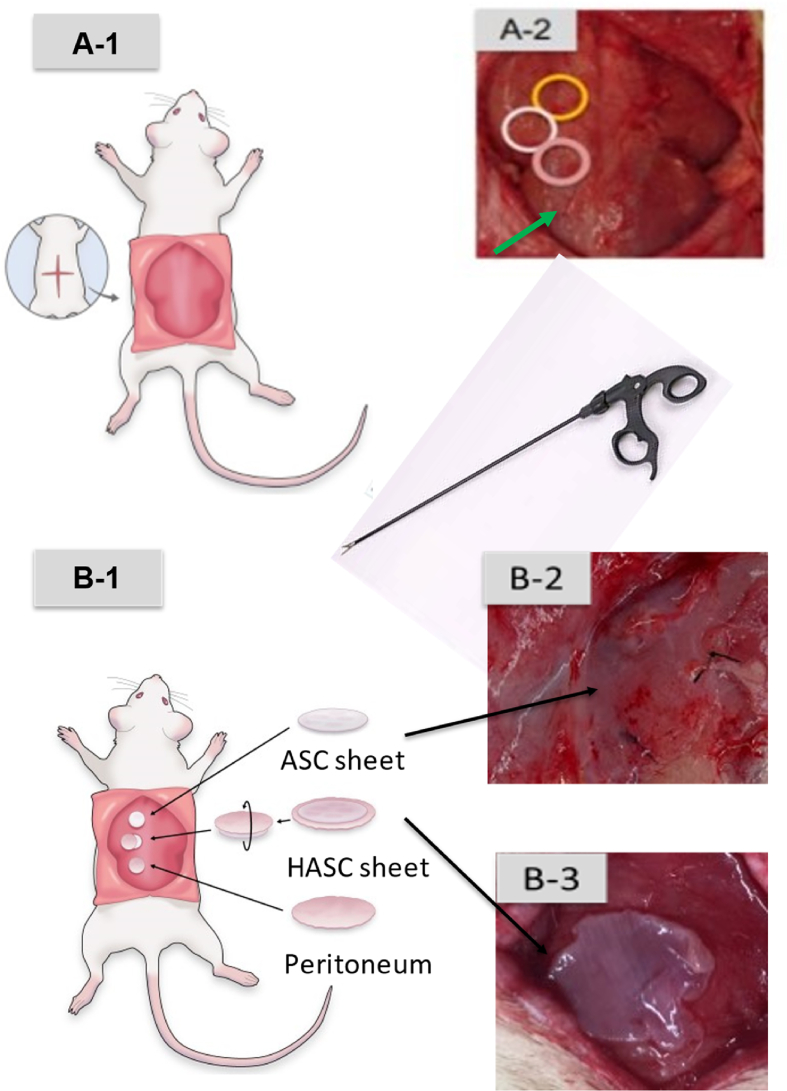
Transplantation of cell sheets. The location of transplant. A cross-incision was made on the dorsal skin, the vastus lateralis muscle was identified, and the fascia was removed. (**A-1, 2**) Sham operation was performed as a control in the area indicated by the arrow. (**A-2**) Adipose-derived stem cell (ASC) sheets, hybrid adipose-derived stem cell (HASC) sheets, and the peritoneum were implanted on the exposed muscle bodies. How they are transplanted. Forceps were used to transplant the ASC sheet over the muscles of the rat's back. (**B-1, 2, 3**) HASC sheet was transplanted upside down so that the ASC sheet was in direct contact with the muscle body. The transplantation sites were marked with sutures.(**B-3**)

The experimental design and procedure for evaluating the transplantation of ASC sheets and HASC sheets onto the latissimus dorsi muscle of SD rats are schematically shown in Figs. 1 and 2, respectively. Approximately 2–3 weeks before transplantation, ASCs were isolated from the subcutaneous fat collected from the inguinal region of SD-Tg rats under inhalation anesthesia with 2–4% isoflurane. ASCs were cultured until passage 3 and were then seeded on 35-mm temperature-responsive dishes (Cellseed Inc., Tokyo, Japan) at a density of 1 × 10^6^ cells/dish. The resulting ASC sheets were harvested from the dishes at 80% confluency by reducing the temperature to 20 °C for 30 min. The ASC sheets thus obtained were layered with the peritoneum extracted from the abdominal wall of the SD rats to form an HASC sheet, which was transplanted on the muscle body on the backs of SD rats following cross-incision and evaluated macroscopically and histopathologically on postoperative days 3 and 7.

### 2.3 Isolation of ASCs

SD-Tg rats were given an inhalant anesthetic with 2.0–4.0% isoflurane. After sterilizing the abdomen, the subcutaneous adipose tissue was surgically excised from the bilateral inguinal region. The extracted tissue was washed with 10 mL phosphate-buffered saline (PBS) and minced into small pieces in 20 mL PBS. The minced tissue was enzymatically digested with 0.1% type-2 collagenase (Worthington Biochemical Corporation, Lakewood, NJ, USA) at 37 °C for 2 h at 130 rpm in the shaking bath (Thamato. Yamato Scientific Co., Ltd. Tokyo, Japan). After filtration by a cell strainer and centrifugation (Ax-310. Tomy, Tokyo, Japan) at 4 °C at xg 700 for 5 min, ASCs were collected as the pellet. The filtration and centrifugation steps were repeated twice. ASCs were seeded at a density of 1 × 10^5^ cells/dish on a 100-mm dish with complete medium (Fujifilm Wako Pure Chemical Corporation) containing 10% fetal bovine serum (FBS; Life Technologies, Frederick, MD, USA) and 1% penicillin/streptomycin (Fujifilm Wako Pre-Chemical Corporation, Osaka, Japan) and cultured at 37 °C with 5% CO_2_.

### 2.4 Culture of ASCs

ASCs were cultured for 7 days with the first replacement of culture medium performed the next day and subsequent replacements performed every 3 days. The cells were passaged upon reaching 80% confluency. For passaging, ASCs were washed twice with PBS and treated with 0.25% (w/v) trypsin and 0.1% (w/v) ethylenediaminetetraacetate, phenol red (Gibco, Thermo Fisher Scientific, Waltham, MA, USA) for 3 min at 37 °C. The disassociated ASCs were collected with 10% FBS/DMEM and centrifuged at 4 °C and 2000 rpm for 5 min. The resulting pellet was resuspended in 10% FBS/DMEM. After counting the total cells, 1 × 10^5^ cells were seeded in a 100-mm dish. ASCs were similarly passaged twice.

### 2.5 Preparation of ASC sheet

At passage 3, ASCs were seeded at a density of 1 × 10^6^ cells/dish on 35-mm-diameter temperature-responsive dishes (UpCell; CellSeed, Tokyo, Japan) and cultured in complete culture medium at 37 °C with 5% CO_2_. The medium was changed to complete medium with 16.4 mg/mL ascorbic acid (Fuji film Wako Pure Chemical Industries) and was replaced every 24 h for an additional 2–3 days until the cells reached 90% confluency. The cells were harvested before reaching 100% confluence to maintain their proliferative capacity after transplantation. ASC sheets were harvested from the dishes by reducing the temperature to 20 °C for 30 min. ASC sheets were detached without any chemical treatment.

### 2.6 Harvested the peritoneum from the abdominal wall

SD rats were anesthetized with 2.0–4.0% isoflurane (Fujifilm Wako Pure Chemical Corporation), followed by laparotomy. The peritoneum was extracted from the abdominal wall and trimmed into a circular shape of 30–35 mm in diameter.

### 2.7 Preparation of hybrid adipose derived stem cell sheet

The trimmed peritoneum was laminated with the ASC sheet, which was grown in the temperature-responsive culture dish (Fig. 1). HASC sheet was so named because of the hybrid nature of layered ASC sheet with the peritoneum.

### 2.8 Transplantation of the cell sheets

A cross-incision was made on the backs of the rats, the subcutaneous tissue and fascia were removed, and three groups of sheets (i.e., ASC sheets, HASC sheets, and the peritoneum) were implanted on the muscle body with laparoscopic forceps (Fig. 2). For all three groups, the incisions were closed with 5-0 nylon thread marking the transplant sites. As a control, a sham operation was also performed. The cross-incision on the back was sewed similarly. The transplanted rats had free access to feed and drinking water in the cage after awaking from anesthesia.

### 2.9 Specimen retrieval from the transplantation site

Because the incidence of colorectal surgery in humans is high within 7 days after surgery [12], depending on when the patient starts eating, the evaluation time was set at days 3 and 7 after surgery, even though the transplantation was performed on the back. Post-transplantation, on days 3 and 7, SD rats were anesthetized with isoflurane. The sutured cross-sectional skin section was opened and the entire muscle of the graft site on the back was retrieved under ultraviolet light.

### 2.10 Histopathological examination

Specimens excised from each rat were rinsed with saline solution. After visual confirmation, the specimens were soaked and fixed in 4% paraformaldehyde (Muto Pure Chemical, Tokyo, Japan) for 2–3 days. The specimens were dissected into small pieces and embedded in paraffin. For histological analysis, 5-mm paraffin-embedded sections were stained with hematoxylin-eosin (HE) using conventional methods. They were also stained with anti-GFP antibody, CD34 and calretinin. Therefore, ASC sheet alone also stained with HE, oil red and CD34. The slides were observed with a Nikon Eclipse E800 Microscope and NISElements (Nikon, Tokyo, Japan).

### 2.11 Statistical analysis

We compared the transplant sites of rats transplanted with ASC sheet and HASC sheet from macroscopic and histological aspects. Spread and attachment of the cell sheets were analyzed based on whether or not the 30-mm diameter sheets shrunk to a diameter of less than 10 mm. The comparison between groups was based on the shorter horizontal and vertical diameters and assessed with the chi-square test; P < 0.01 was considered statistically significant.

## 3 Results

### 3.1 Transplantation procedure

All transplantation experiments were conducted using laparoscopic forceps. As expected, ASC sheet was too soft and fragile to transplant without the use of a carrier. Conversely, HASC sheet was sufficiently stiff to transplant with ease. The difficulty in transplantation using laparoscopic forceps was found to be equivalent to or more difficult than that using surgical tweezers.

### 3.2 Macroscopic findings

**Fig. 3:**
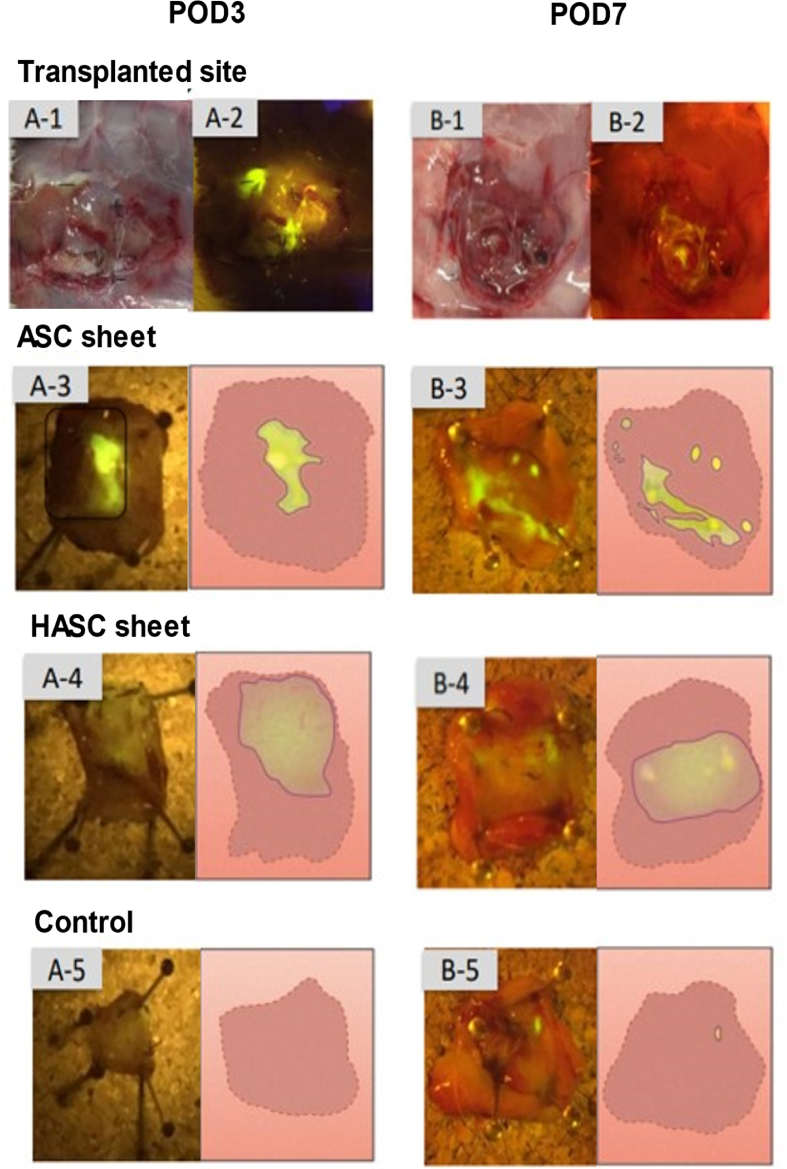
Post transplantation of ASC sheet and HASC sheet. On days 3 and 7 post-transplantation, The specimens of implant sites wzs represented macroscopically. (**A-1, B-1**) The transplant site were examined under ultraviolet. Images representing specimens retrieved on days 3 and 7 post-transplantation are labeled (**A-1, B-1**) Adipose-derived stem cells (ASC) sheets were shrunken. (**A-3, B-3**). Hybrid adipose-derived stem cells (HASC) sheets were faintly transplanted in a spreading condition.(**A-4, B-4**) The control group were no ASC cells, the implanted site does not glow fluorescently.(**A-5, B-5**)

**Fig. 4:**
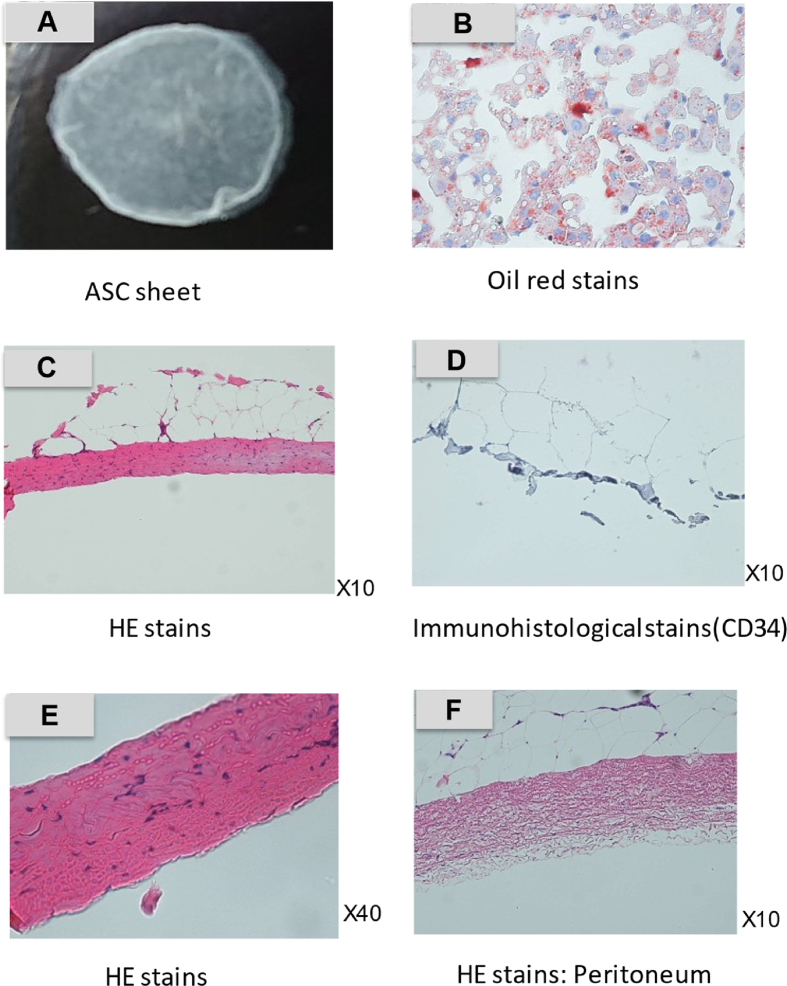
Histopathological findings of ASC sheet and peritoneum. This is ASC sheet before transplanting.(**A**) Cultured ASC sheet alone was stained with HE (**C, E**) and oil red stains (**B**). HE stains showed cell proliferation in the sheet-like tissue (**C, E**). Oil red staining showed red-stained adipocytes in ASC sheet (B). It was also stained with immunohistopathological marker, CD34 (**D**). The CD34 hematopoietic marker showed a negative reaction. HE stains of the peritoneum showed mesothelial cells within a fibrous thickening with gaps. (**F**).

**Fig. 5:**
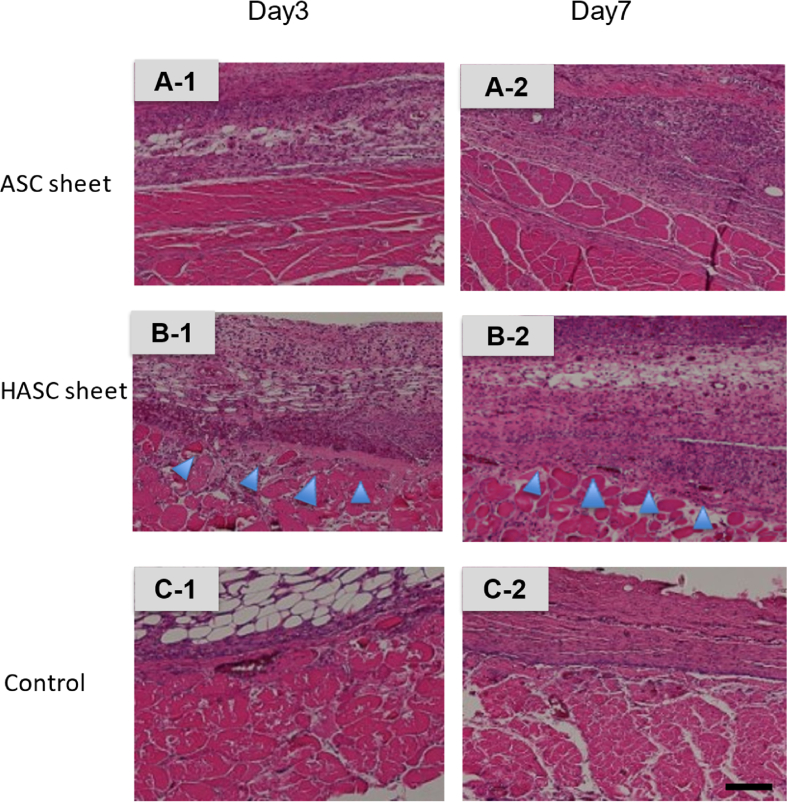
Histopathological findings of transplanted location. Three groups, adipose-derived stem cell (ASC) sheets (**A-1, 2**), hybrid adipose-derived stem cells (HASCs) sheets (**B-1, 2**), and control (**C-1, 2**), stained differently with hematoxylin-eosin. ASC sheets were found to be shrunken in the specimens retrieved from the transplantation sites on days 3 and 7 post-transplantation. (**A-1, 2**) In contrast, HASC sheets spread extensively.( **B-1, 2**)

We hypothesized that ASC sheet would be hard to handle for laparoscopic operations and that the peritoneum would be an effective carrier for transplantation. ASC sheets were found in shrunken states in the specimens retrieved on day 3 as well as on day 7 post-transplantation (Fig. 3 A-3, B-3). By contrast, the HASC sheets remained attached in an expanded state in specimens retrieved on both days 3 and 7 post-transplantation (Fig. 3A-4, B-4). In the control group, no cell sheet or contamination was observed (Fig. 3 A-5, B-5).

### 3.3 Histopathological findings

ASC sheet and harvested peritoneum evaluated histopathologically by HE stains (X10, X40), prior to transplantation (Fig. 4). HE stains showed adipocytes and sheet-like aggregated cell proliferation. Oil red staining showed adipocytes stained red and cell proliferation during differentiation (Fig. 4).

We compared transplanted ASC sheets and HASC sheets using HE stains (x40). Transplanted ASC sheet is found cell proliferation locally. In contrast, ASC sheet is found widespread cell proliferation with peritoneal tissue (Fig. 5).

### 3.4 Spread and attachment of cell sheets

It is critical to ensure that cell sheets are effectively spread out and attached. We quantitatively compared how well the ASC sheets alone and HASC sheets spread on days 3 (Table 1) and 7 (Table 2) post-transplantation based on image analysis (Fig. 3. A-3,4, B-3,4). The point estimate of the odds ratio was considered to be statistically significant at P < 0.01 for both days based on the chi-square test.

**Table 1:** Quantitative analysis of the spread of adipose-derived stem cell (ASC) and hybrid adipose-derived stem cell (HASC) sheets at day 3 post-transplant.

Sheet	Diameter <1 cm or disappeared	Diameter >1 cm	Total
ASC sheet	8	2	10
HASC sheet	0	10	10
Total	8	12	20

**Table 2:** Quantitative analysis of the spread of adipose-derived stem cell (ASC) sheets and hybrid adipose-derived stem cell (HASC) sheets at day 7 post-transplant.

Sheet	Diameter <1 cm or disappeared	Diameter >1 cm	Total
ASC sheet	9	1	10
HASC sheet	1	9	10
Total	10	10	20

**Fig. 6:**
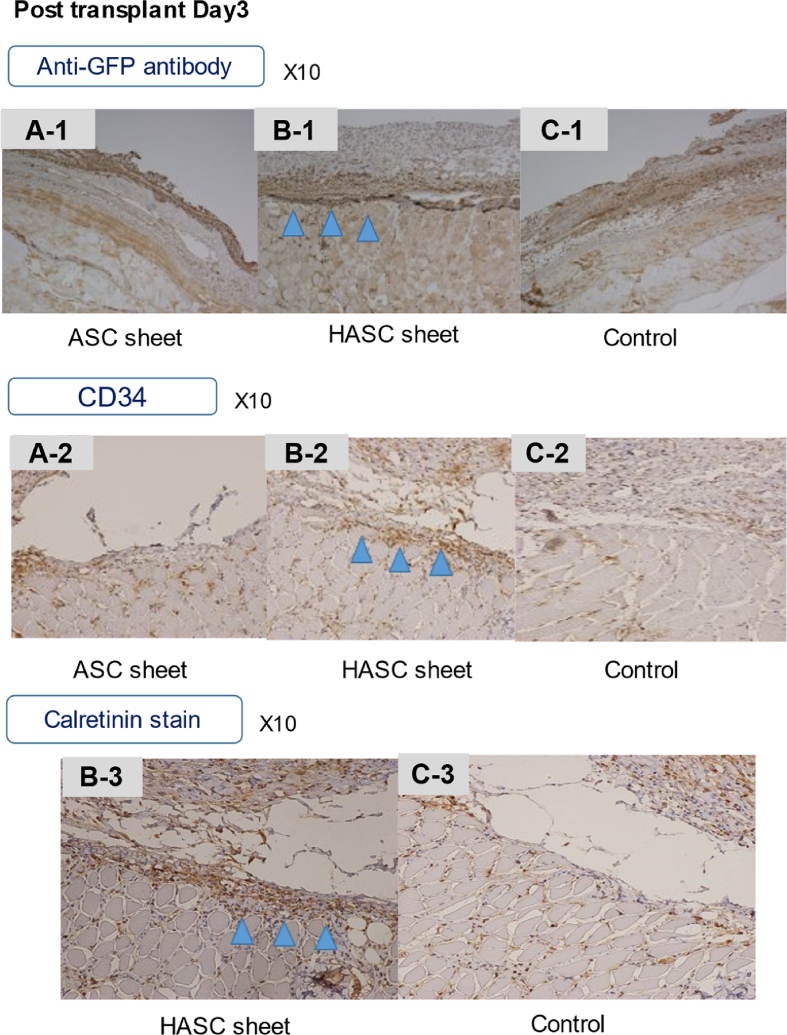
Immunohistopathological findings of transplant location. Immunostaining with anti-GFP antibody obscured the transplanted area in the ASC sheet group (**A-1**). In contrast, HASCs sheets showed fibrous thickening and cell proliferation at the site of implantation directly in the muscle layer (**B-1**). Immunostaining with CD34 was negative in the ASC sheet group (**A-2**). The HASC sheet group showed very slight staining of hematopoietic cells, but this was not a clear indication of sheet-induced cell proliferation (**B-2**). Calretinin staining is an immunostaining that stains for calcium-binding proteins, but was not performed on the ASC alone group, but on the HASC sheet group (**B-3**), because a positive reaction was expected in the mesothelial cells. HASCs sheets reacted slightly more positively than the control group (**C-3**), indicating widespread cell proliferation in and around the area.

## 4 Discussion

Tissue engineering and regenerative medicine have evolved with recent biotechnological advances, which combine biomaterials, growth factors, and stem cells to repair failing organs and promote wound healing [3,13,14]. Several conditions such as organ failure, tissue loss due to trauma, cancer abrasion, and congenital structural anomalies, can be treated by clinical procedures/surgical strategies, including organ transplantation, autologous tissue transfer, and the use of artificial materials. However, these treatments have potential limitations, including organ shortages, damage to healthy parts of the body during treatment, allergic reactions, and immune rejection [15]. Cell-sheet engineering technology is one of the major advances in the field of tissue engineering. Cell-sheet engineering involves culturing cells and harvesting them into a sheet. The advantages of a cell sheet are rapid cell engraftment, no immune rejection owing to the use of autologous transplantation, and improved quality of life without impairing any residual function [16]. Compared to the injection of a cell suspension, engineered cell sheets have advantages that include the retention of cells at the transplantation site for a longer period of time and the local delivery of growth factors and cytokines [17]. The most important aspect of cell sheet engineering is the existence of temperature-responsive culture dishes. Okano et al. developed a temperature-responsive culture dish that was made of poly (N-isopropylacrylamide) (PIPAAm), a hermos-responsive polymer, which has a low critical solution temperature of approximately 32 °C in water [1]. Yoshida et al. also reported that PIPAAm is soluble in aqueous media at solution temperatures below 32 °C, which is its critical solution temperature [[1], [18], [19]]. Temperature-responsive culture dishes on which a temperature-responsive polymer, PIPAAm, is covalently immobilized, enable noninvasive harvesting of cultured cells and the fabrication of transplantable cell sheets [19,20]. By covalently immobilizing PIPAAm onto ordinary tissue culture polystyrene surfaces (TCPS) at nanometer-scale thickness, cell adhesion and detachment can be controlled by effortless temperature changes [19]. On these surfaces, various cell types adhere, spread, and proliferate similar to that on normal TCPS at 37 °C. However, by reducing the incubation temperature to 20 °C, all cultured cells can be spontaneously detached owing to the conversion of the grafted PIPAAm from hydrophobic to hydrophilic [[21], [22], [23], [24]]. Cell sheet preparation using this temperature-responsive culture dish can be performed on various types of cells [24], including keratinocytes, skeletal myoblast cells [25], MSCs, and ASCs [26].

Cell sheet engineering has progressed in recent years, and reconstruction techniques following the removal of malignant tumors such as esophageal or stomach cancers using oral mucosal epithelial cell sheets prepared with the patient's own cells are gradually being used in clinical practice [27]. In the field of ophthalmology, Nishida et al. reported that tissue-engineered cell sheets from autologous ora mucosal epithelium served as effective substitutes for allografts of limbal tissue in reconstruction of corneal and limbal surfaces without serious complications [4].

In the field of cardiovascular surgery, myoblast sheets produce cytokines such as hepatocyte growth factor, which may have a positive impact on the c-Met-expressing damaged myocardium, thus leading to the attenuation of fibrosis, angiogenesis, and recruitment of stem cells induced by paracrine cytokines. Autologous myoblast cell sheet transplantation may positively contribute to the improvement of the clinical condition of patients with dilated cardiomyopathy (DCM), allow the discontinuation of a left ventricular assist system (LVAS), and avoid heart transplantation. Therefore, this therapy shows promise for myocardial regeneration in patients with end-stage DCM [25]. Miyagawa et al. also studied autologous skeletal stem-cell sheets to evaluate their safety and feasibility as the sole therapy for patients with severe heart failure who had already received the maximum medical treatment available [28]. They applied skeletal stem-cell sheet therapy to idiopathic dilated cardiomyopathy. The same group previously reported that in a hamster model of DCM, cell-sheet implants preserved functional performance and attenuated dilation of the left ventricle chamber [29].

In the field of dentistry, cryotherapies combining stem cell biology and tissue engineering offer a promising approach for overcoming these limitations. The safety and efficacy of autologous periodontal ligament-derived cell sheets in severe periodontal defects were evaluated, along with the stability of this efficacy during mid-long-term follow treatment of severe periodontal defects. Another study reported that cone beam computed tomography is useful for measuring periodontal defects [30].

In the field of otolaryngology, cell sheet transplantation around the stapes was performed using a syringe probe device, and a tympanic membrane was fashioned using a cartilage/cell sheet hybrid. Recurrence of tympanic membrane adhesion was prevented by grafting the cell sheet around the stapes and to the rear surface of the newly fashioned tympanic membrane [31,32]. Hama et al. reported cell sheets created from the nasal mucosa, which is easier to access than the middle ear cavity, which were. useful as a xenograft material in mucosa reproduction after a middle ear operation [33].

The placement of cell sheets during transplantation had a direct effect on the location of epithelialization without scar stenosis [5,6]. The transplanted cells could serve as the cell source of regenerated epithelia, but it is also possible that wound healing is promoted by growth factors and cytokines secreted by transplanted cell sheets.

Similarly, stem cell therapy harnessing the effect of immune regulation ability as well as the tissue-repairing properties of MSCs has been developed for the treatment of intractable diseases [34,35]. MSCs have the ability to undergo self-renewal and differentiate along multiple lineage pathways [35]. We have conducted basic experiments to utilize such cell sheet technology for the enhancement of gastrointestinal anastomosis, which we plan to implement in the future. In this study, we focused on MSCs, which. are multipotent cells capable of differentiating into a variety of specialized cells, such as osteoblasts, chondrocytes, and adipocytes [8]. The adipose tissue contains a large number of MSCs.

ASCs are differentiated through complex processes accompanied by coordinated changes in cell morphology, hormone sensitivity, and gene expression [35]. ASCs offer several advantages in regenerative medicine. They are easily isolated, capable of paracrine activity such as local immune modulation, cell recruitment, and neovascularization. They can be able to differentiate into different cell types such as mesenchymal and non-mesenchymal lineage [36]. The ability of ASCs to secrete growth factors and cytokines offer further advantages in promoting wound healing or improving the quality of tissues that are regenerated [35]. Wound healing is a complex process that involves the coordinated efforts of many types of cells and the cytokines they release. The role of ASCs in wound healing and tissue regeneration has provided new options for treating difficult wounds [11].

Several reports indicate that ASCs help to ameliorate tissue inflammation and can accelerate new blood vessel formation [[35], [36]]. The beneficial characteristics of ASCs could be exploited to promote the healing process after surgery, thus help preventing postoperative complications [36]. The therapeutic potential of ASC sheets has been demonstrated, ASC sheet, could improve cardiac tissue regeneration in the treatment of myocardial infarction [37], DCM [38], healing of hind limb ischemia [39], and chronic non-healing skin wounds [40]. The beneficial characteristics of ASCs could also be exploited to promote the healing process after surgery, thus helping to prevent postoperative complications [[12], [26]]. ASC sheets could provide local delivery of cells to enhance healing and prevent leakage.

One of the common and the most serious post-operative complications is an anastomotic leakage [[26], [41]], which may occur because of technical issues, the pressure of anastomosis, blood flow problems, prolonged inflammation, and infection. Although various measures are taken to prevent complications such as pretreatment, bowel lavage, additional sutures, and stoma placement, leakage occurs in 1–10% of rectal surgeries in Japan. Globally, leak rates vary between 1% and 19% depending on the site of the anastomosis, with 20–30% of affected patients dying as a result of the complication [[26], [41], [42]]. Although anastomotic leakage in the colon is quite rare, it is quite frequent in the lower rectum. During lower rectal surgery, stoma placement is often performed. Currently, the use of preoperative chemoradiation is increasing, and stoma placement is often performed to prevent anastomotic leakage. Although this is a useful method to prevent anastomotic leakage, it decreases the quality of life of the patient.

ASC sheets have been shown to be effective in promoting the wound-healing process [35]. Therefore, ASC sheets are considered to be effective for anastomotic leakage. A clinical study on the treatment of radiation-induced tissue damage using human ASCs showed progressive improvement in tissue hydration and new vessel formation [11]. A previous study reported that the cell sheet engineering technique was effective for anastomotic leakage in animal models. Nakamura et al. used human skeletal muscle myoblast sheets [43], and Burke et al. reported that the delivery of MSCs to an anastomosis in an animal model was safe and feasible [44].

Although ASCs offer several advantages in regenerative medicine [[26], [45]], ASC sheets are difficult to apply to clinical medicine. Laparoscopic and robotic surgery is used in multiple surgical subspecialties, including urology, gynecology, surgical oncology, bariatric and foregut surgery, colorectal surgery, and cardiac and thoracic surgery [46], and is becoming increasingly common in the field of lower gastrointestinal surgery. The ASC sheet is very soft and brittle. Although it is difficult to insert these sheets into the abdominal cavity and place them on the anastomotic site with laparoscopic or robotic forceps, it is necessary to spread them during transplantation.

Therefore, we considered the application of the peritoneum as a support. The peritoneum is a biological membrane in the abdominal cavity with a network of small blood vessels running through it. Peritoneal fibrinolysis seems to be an important denominator in the early formation of post-surgical adhesions [47]. Fibrin is formed at injured sites either from bleeding or by post-traumatic inflammatory mechanisms, such as leakage from the vasculature caused by vasoactive substances (histamine), or other mediators released from recruited white blood cells, and direct fibrin formation from the peritoneal fluid [48]. The response of the peritoneum to infective organisms involves inflammatory cytokines and the interaction between resident cell populations, i.e., macrophages, mesothelial cells, and fibroblasts [49]. The peritoneum consists of a layer of mesothelial cells and underlying connective tissue. As a biological membrane, it has physiological functions such as exudation, leakage, and secretion, and serves as a defense mechanism against infection. The omentum, which is a type of a peritoneum, is yellowish-brown in color due to the accumulation of adipose tissue, lymphocytes, and plasma cells, and has rich mobility that prevents the spread of inflammation. The omentum contains fat, capillaries, lymph vessels, endothelial cell spaces, and water vacuoles. The advantages of using the peritoneum are that it can be easily harvested during surgery for autologous transplantation and prevents the progression of inflammation. Therefore, the peritoneum is not only easy to handle but also physiologically relevant. At present, several synthetic materials have been made commercially available to be used as supports; however, there is a risk of infection due to the synthetic nature of the products that require collection or even those that do not require collection. Therefore, the hypothesis for the present study, i.e., using the peritoneum, which can be harvested during surgery and is not easily affected by the immune response due to autologous transplantation, as a support.

In this study, we developed an HASC sheet by layering the ASC sheet and the peritoneum, evaluated its feasibility. ASC and HASC sheets were compared in terms of ease of transplantation using laparoscopic forceps and spread post-transplantation. ASC sheets were difficult to properly implant in a spread-out position covering the anastomosis with forceps. In macroscopic findings, on days 3 and 7 post-transplantation, ASC sheets were found to have shrunk, whereas HASC sheets were well-spread. In histopathological findings, the peritoneum and proliferated ASCs were found in HASCs. There was a significant difference between the spread of ASC and HASC sheets. Thus, our experiments demonstrated that hybrid cell sheets can be ectopically transplanted with the autologous peritoneum as a support membrane. The HASC sheet is easy and effective to transplant with laparoscopic forceps and has potential application in clinical medicine in gastroenterological surgery.

This study has several limitations. As anastomotic leakage is common in the lower rectum, the site of the sheet transplantation should be at the anastomosis of the lower rectum. This experiment used rats, which made it difficult to compare and examine the results in an anastomotic model and apply the sheet to the lower rectum in the pelvis. The back of the body was chosen as the transplantation site because the lower rectum exists without the peritoneum, although there is an anatomical difference between the smooth muscle and skeletal muscle. Therefore, in this study, we transplanted the sheets to the back muscles of the body. From an animal welfare perspective, the number of rats was kept to a minimum, resulting in a small number of transplantations.

We used CD34, a hematopoietic factor, as a marker to stain ASC sheet. The result was negative. We should use more mesenchymal stem cell-specific markers should have been used such as CD105, 106, Thy-1 and so on. The results of staining the grafts with CD34 showed slight staining, but more cell-specific markers should have been used as well.

Therefore, cytological, and immunological evaluations were not performed in this study because the study was focused on the handling of the sheets during the transplantation. In other words, while we understand that various other studies have shown that ASCs are cytologically superior, our study was only conducted to develop HASC sheets as a device that would allow ASCs to be more easily and accurately transplanted in the difficult transplantation situation of lower gastrointestinal tract surgery, and we made no mention of the cytological superiority of HASCs. We hope to examine whether HASC is also cytologically superior in future studies.

## 5 Conclusions

The HASC sheet, which consists of layers of ASC sheet and autologous peritoneum, is easy to handle, can be transplanted precisely, and has immense therapeutic potential for clinical application.

## Author contribution statement

Natsuki Matsuo: Performed the experiments; Wrote the paper.

Takeshi Ohki: Conceived and designed the experiments; Analyzed and interpreted the data; Wrote the paper.

Shota Aoyama: Performed the experiments.

Shigeki Yamaguchi; Michio Itabashi; Hiroto Egawa: Contributed reagents, materials, analysis tools or data.

Masakazu Yamamoto: Conceived and designed the experiments.

## Funding statement

This study was supported by Grant-in-Aid for Scientific Research (C) KAKENHI from the Japan Society for the Promotion of Science (JSPS) (Grant no: JP18K0855).

## Data availability statement

Data included in article/supp. material/referenced in article.

## Declaration of interest’s statement

The authors declare no competing interests.
